# Cuproptosis, the novel type of oxidation-induced cell death in thoracic cancers: can it enhance the success of immunotherapy?

**DOI:** 10.1186/s12964-024-01743-2

**Published:** 2024-07-27

**Authors:** Ruiwen Zhao, Olga Sukocheva, Edmund Tse, Margarita Neganova, Yulia Aleksandrova, Yufei Zheng, Hao Gu, Deyao Zhao, SabbaRao V. Madhunapantula, Xiaorong Zhu, Junqi Liu, Ruitai Fan

**Affiliations:** 1 The Department of Radiation Oncology & Cancer Center, The First Affiliated Hospital of Zhengzhou University, Zhengzhou, 450052 China; 2 Department of Gastroenterology and Hepatology, Royal Adelaide Hospital, Port Rd, Adelaide, SA 5000 Australia; 3 Nesmeyanov Institute of Organoelement Compounds, Russian Academy of Sciences, Moscow, 119991 Russia; 4 Special Interest Group in Cancer Biology and Cancer Stem Cells (SIG-CBCSC), Department of Biochemistry, JSS Medical College, JSS Academy of Higher Education & Research, Mysuru, Karnataka 570015 India

**Keywords:** Cancer therapy, Cuproptosis, Ferredoxin 1 (Fdx1), Tumor microenvironment (TME), Oxidative stress, Immunoediting

## Abstract

Copper is an important metal micronutrient, required for the balanced growth and normal physiological functions of human organism. Copper-related toxicity and dysbalanced metabolism were associated with the disruption of intracellular respiration and the development of various diseases, including cancer. Notably, copper-induced cell death was defined as cuproptosis which was also observed in malignant cells, representing an attractive anti-cancer instrument. Excess of intracellular copper leads to the aggregation of lipoylation proteins and toxic stress, ultimately resulting in the activation of cell death. Differential expression of cuproptosis-related genes was detected in normal and malignant tissues. Cuproptosis-related genes were also linked to the regulation of oxidative stress, immune cell responses, and composition of tumor microenvironment. Activation of cuproptosis was associated with increased expression of redox-metabolism-regulating genes, such as ferredoxin 1 (FDX1), lipoic acid synthetase (LIAS), lipoyltransferase 1 (LIPT1), dihydrolipoamide dehydrogenase (DLD), drolipoamide S-acetyltransferase (DLAT), pyruvate dehydrogenase E1 subunit alpha 1 (PDHA1), and pyruvate dehydrogenase E1 subunit beta (PDHB)). Accordingly, copper-activated network was suggested as an attractive target in cancer therapy. Mechanisms of cuproptosis and regulation of cuproptosis-related genes in different cancers and tumor microenvironment are discussed in this study. The analysis of current findings indicates that therapeutic regulation of copper signaling, and activation of cuproptosis-related targets may provide an effective tool for the improvement of immunotherapy regimens.

**Graphical Abstract:**

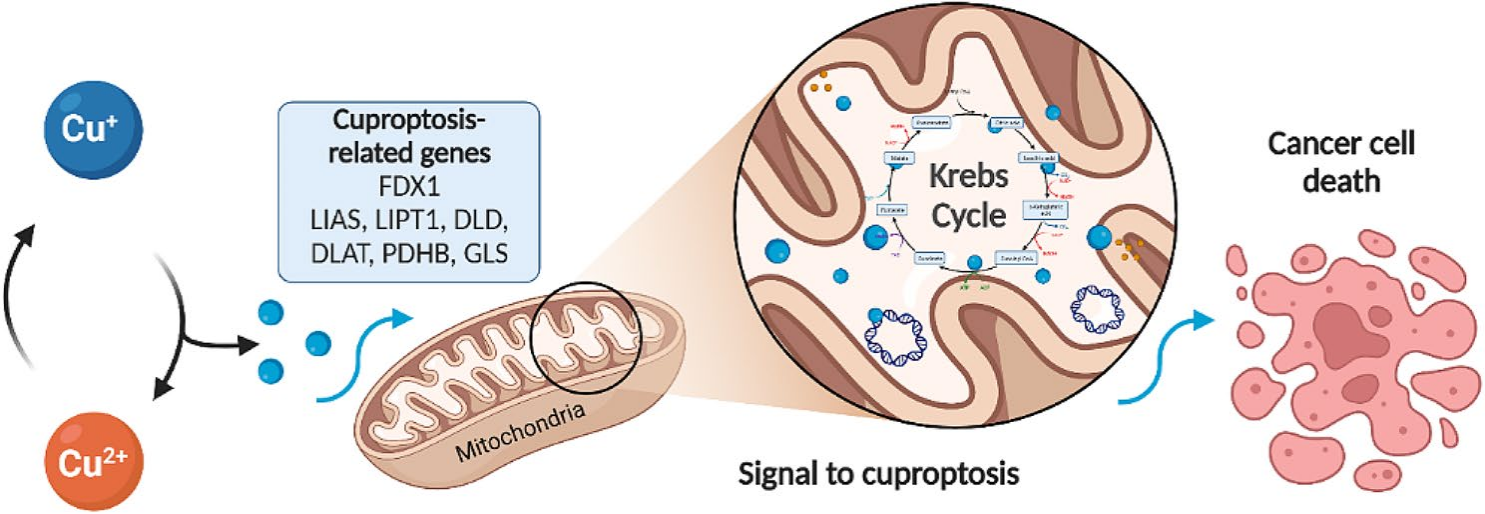

## Key messages

**Facts**

• Copper ions, essential components of human body, can activate a novel subtype of programmed cell death, defined as cuproptosis.

• Cuproptosis is tightly associated with the regulation of mitochondrial respiration and oxidative stress in various cells, including malignant.

• Changed concentration of copper ions was found to regulate a set of redox-metabolism-regulating genes, such as ferredoxin 1, lipoic acid synthetase, lipoyltransferase 1, dihydrolipoamide dehydrogenase, drolipoamide S-acetyltransferase, and pyruvate dehydrogenase E1 subunits alpha 1 and beta.

• Cuproptosis-related genes and copper-containing compounds were indicated as potential targets and tools for the development of novel anti-cancer therapy.

**Open Questions**

• How to target cuproptosis-related genes to stop cancer cell survival?

• Which copper-responsive targets and/or copper-containing compounds can be employed safely and effectively during cancer treatment?

• Which biomarkers can be reliably employed to define good response to the activation of cuproptosis in cancer cells?

• Are there others, undiscovered intracellular targets of copper which can be used for cancer prevention and treatment?

## Introduction

Metal element copper is required for the effective functioning of human organisms. Copper is used as a micronutrient involved in several important catalytic processes as a structural cofactor of metal-dependent enzymes [1, 2]. The presence of this element is required for the metabolic regulation of growth and functional activities of the human body [3, 4]. The normal copper concentration, which is equal to about 1 mg/L in blood plasma and 50–120 mg for the total body content in average adults, is beneficial for the maintenance of proper homeostasis, while high or low concentrations of copper may be damaging for the optimal physiological state [5, 6]. Copper deficiency has been linked to diseases including anemia [7], osteoporosis [8], obesity [9], coronary heart diseases [10, 11], and cancers [6, 12]. Disorders of copper metabolism can also cause neurological pathologies, including Wilson’s [13], Alzheimer’s [14, 15], and Parkinson’s diseases [16–18]. A high concentration of copper is a cytotoxic, cell death-activating factor [19, 20]. Copper-induced cell death was defined as cuproptosis (terminology suggested by Tsvetkov [21]), a novel form of programmed cell death (PCD) that is different from apoptosis, necroptosis, pyroptosis, and ferroptosis [21, 22]. For instance, apoptosis is a classical PCD type which is marked by activation of several death pathways, chromatin condensation, caspase (-1, -3 and − 8) cleavage, and release of cytochrome c (cyt c) from mitochondria. Various internal physiological factors, such as cytokines and glucocorticoid hormones, can trigger PCD gene activation in apoptosis [23, 24]. The mitochondrion is the central intracellular organelle which is responsible for the propagation of classical apoptosis. Mitochondria are also affected during ferroptosis and cuproptosis.

Unlike apoptosis, chromatin condensation and/or caspase − 3 cleavage were not reported during activation of ferroptosis or cuproptosis [25, 26]. Ferroptosis is a PCD trigged by the accumulation of iron ions, dysregulated iron metabolism, modified activation of specific iron-activated genes responsible for lipid synthesis and peroxidation [25]. Ferroptosis is accompanied by the functional failure or blockade of the glutathione-dependent/antioxidant cell defense system. Both ferroptosis and cuproptosis are activated by excessive retaining of metal ions (iron and copper, respectively), marked by the shrinking of mitochondria, disrupted mitochondrial membrane, and abnormal energy metabolism [21, 23, 27, 28]. While apoptosis is a PCD required for developmental programs in multicellular organisms, cuproptosis and ferroptosis were defined as metabolic types of PCD, which are not yet linked to tissue- and organogenesis [25, 26]. Accenting the differences, the inhibitors of ferroptosis and apoptosis do not block activation of cuproptosis. Furthermore, while ferroptosis is mainly induced by disturbances in cell defense against abnormal lipid peroxidation, cuproptosis can be directly activated by the excessive amount of intracellular copper which is tightly associated with the regulation of mitochondrial respiration. In mitochondria, copper causes the aggregation of lipoylation proteins and the loss of iron-sulfur (Fe-S) cluster proteins [27]. These processes are mediated by the binding of copper ions to the lipoylated tricarboxylic acid (TCA), resulting in increased proteotoxic stress and cell destruction [2, 21, 27]. Cuproptosis can be modulated by copper ion carriers, such as elesclomol, and copper chelators [27, 28].

The current study reviews recent advances in the understanding of cuproptosis and copper-associated signaling. We critically discuss the activation of cuproptosis-related genes and their diagnostic values for the selection of targeted cancer treatment. This study is focused on the role of cuproptosis and associated gene signaling in various gastrointestinal cancers. Analysis of recent reports indicates that cuproptosis-related genes and copper-containing compounds [29] can be used as potential targets and tools for the development of novel anti-cancer therapy. Therapy-developing insights and deciphered mechanisms of cuproptosis are presented and discussed.

## Disbalanced copper homeostasis, transport, and role in tumorigenesis

Intracellular copper concentration is very low and tightly regulated in normal cells [30]. Excessive amounts of copper trigger cytotoxicity and copper-associated death of normal cells (defined as cuproptosis) [29, 31]. Copper ions are transported by soluble carrier proteins, copper chaperones [32], which can bind a group of membrane-bound enzymes, including copper exporter ATP7A (Menkes ATPase; a proliferation-regulating effector) [33]. Ionic copper binds divalent metal transporter 1 (DMT1) which reduces Cu^2+^ to Cu^+^ in the intestinal lumen. The reduced copper can be associated with the copper transporter 1 (CTR1; also known as SLC31A1 (solute carrier family 31 member 1)) which delivers copper to the cell cytoplasm. Intracellular copper binds the copper chaperone protein antioxidant 1 (Atox1), which can form a complex with the copper transport adenosine triphosphatase (ATPase) 7B (ATP7B) in the Golgi complex [34, 35]. The chain of reactions facilitates the production of ceruloplasmin (the copper transporter) from pre-ceruloplasmin [20, 36]. Copper-binding ceruloplasmin may be targeted to increase oxidative stress and promote cancer cell death [37].

Significant changes in the concentration of intracellular copper were linked to various pathological conditions. Transformed copper metabolism was found associated with tumorigenesis, dysregulated cell proliferation, induction of tumor microenvironment (TME), metastasis, angiogenesis, and cancer immunoediting [38–40]. Excessive amounts of intracellular copper have been detected in various cancers, including breast [41], prostate [42], colon [43], lung [44], brain [45], liver [46], head and neck [47], and endometrial [48] malignancies. Moreover, the accumulation of copper in tumor cells was also associated with the development of drug resistance [49, 50], indicating the transformation of copper metabolism and signaling in malignant tissues. Copper storage protein metallothionein (MT), which is the established target for the anticancer drug cisplatin [51], can be used by cancer cell to safely sequester this metal ion. However, the role of MT in copper metabolism and resistance to cuproptosis remains to be confirmed.

Cancer recurrence and drug resistance represent two main impediments to the successful eradication of cancers [52]. The development of drug resistance is supported by cancer immunoediting which allows tumor cells to escape from immune surveillance [53]. Copper, as a cofactor and catalytic element in key metabolic and redox enzymes, is involved in the regulation of immune responses [39, 40, 54]. Moreover, copper was associated with the regulation of blood clotting, hormonal processing, and cellular energy metabolism. For instance, the accumulation of lipid-acylated proteins in mitochondria can be triggered by intracellular copper which impacts Fe-S cluster proteins metabolism [1, 55]. Notably, both beneficial and damaging effects were linked to copper-related signaling which is mediated by several distinct molecular mechanisms. Among the most investigated mechanisms is signaling via copper-binding enzymes. The element is required for proper functional activity of Cu, Zn-superoxide dismutases (SODs) SOD1 and SOD3 [56]. Cytochrome c oxidase (COX) and NADH deoxygenase-2 (ND2) also require copper [57].

The discovery of cuproptosis, a new type of cell death, opens new horizons for cancer therapy via targeting cuproptosis-related genes and proteins [29, 58, 59]. The cuproptosis was registered in different cancer cells by independent investigators [60–63], confirming the promising anti-cancer effect of copper. Therefore, cuproptosis-related genes were assessed to detect the association between copper-based treatment and clinical outcome, the characteristics of TME, and immune responses. It has been found that cuproptosis-related genes may serve as important predictive markers [49], although further assessment is warranted. Moreover, not only the gene expression changes should be assessed, but also the protein content and/or enzymatic activities require detailed verification in future cuproptosis studies. Genetic mechanisms of signaling, genes, and relevant signaling pathways which were found to be activated or silenced by excessive amounts of intracellular copper will be discussed below.

## Cuproptosis-related genes and oxidative stress

The cell toxicity of free copper is triggered during the Fenton reaction which leads to the generation of large amounts of reactive oxygen species (ROS) [64]. Therefore, copper activates oxidative stress and different enzymes responsible for cell defense against ROS. Several recent studies assessed cuproptosis-related gene activation patterns [21, 65]. Database screening resulted in the discovery of 13 genes that are associated with cuproptosis [65]. Seven regulatory genes (ferredoxin 1 (FDX1), lipoic acid synthetase (LIAS), lipoyltransferase 1 (LIPT1), dihydrolipoamide dehydrogenase (DLD), drolipoamide S-acetyltransferase (DLAT), pyruvate dehydrogenase E1 subunit alpha 1 (PDHA1), and pyruvate dehydrogenase E1 subunit beta (PDHB)) were upregulated during cuproptosis (positive/copper-induced regulatory mechanisms). Three regulatory genes (metal-regulatory transcription factor-1 (MTF1), glutaminase (GLS), and cyclin-dependent kinase inhibitor 2 A (CDKN2A)) were silenced during cuproptosis (negative regulation pattern) [21]. Furthermore, three copper transporters were found involved in the regulation of cuproptosis, including SLC31A1, ATP7A, and ATP7B [29, 65]. The demonstrated genetic activities warrant protein-based investigations to confirm the correlation between changes in transcripts, associated targets, and end-products of the downstream enzymatic reactions.

The mechanism of cuproptosis is mediated by FDX1 (a reductase), an essential member of the redox-regulating system [66]. Reducing Cu^2+^, FDX1 can generate more toxic Cu^+^, leading to the activation of cellular stress and cuproptosis. The enzyme can promote the lipoylation of proteins in tricarboxylic acid (TCA) cycle, leading to the reduction of Fe-S cluster proteins [67]. The excessive amount of copper and/or overactivated/overexpressed FDX1 may lead to increased amount of toxic Cu + ions which promote metabolic injuries, although this process requires experimental confirmation. Moreover, the process is more complex and may involve other enzymes in mitochondria. An important component of the mitochondrial aerobic respiratory process, the PDH complex includes multiple copies of three enzymes (DLAT, PDHA1, and PDHB) which also regulate lipoylation, the highly conserved lysine posttranslational modification. PDH complex catalyzes oxidation of pyruvate and its conversion into acetyl-CoA prior to its utilization in TCA cycle; thus, connecting the anaerobic process of glycolysis and the oxidative phosphorylation. During activation of cuproptosis and proteotoxic stress, the increased amount of Cu + binds to lipoylated components of PDH (like DLAT), resulting in the aggregation of lipoylated proteins and destabilization of Fe–S protein clusters [67].

**Fig. 1:**
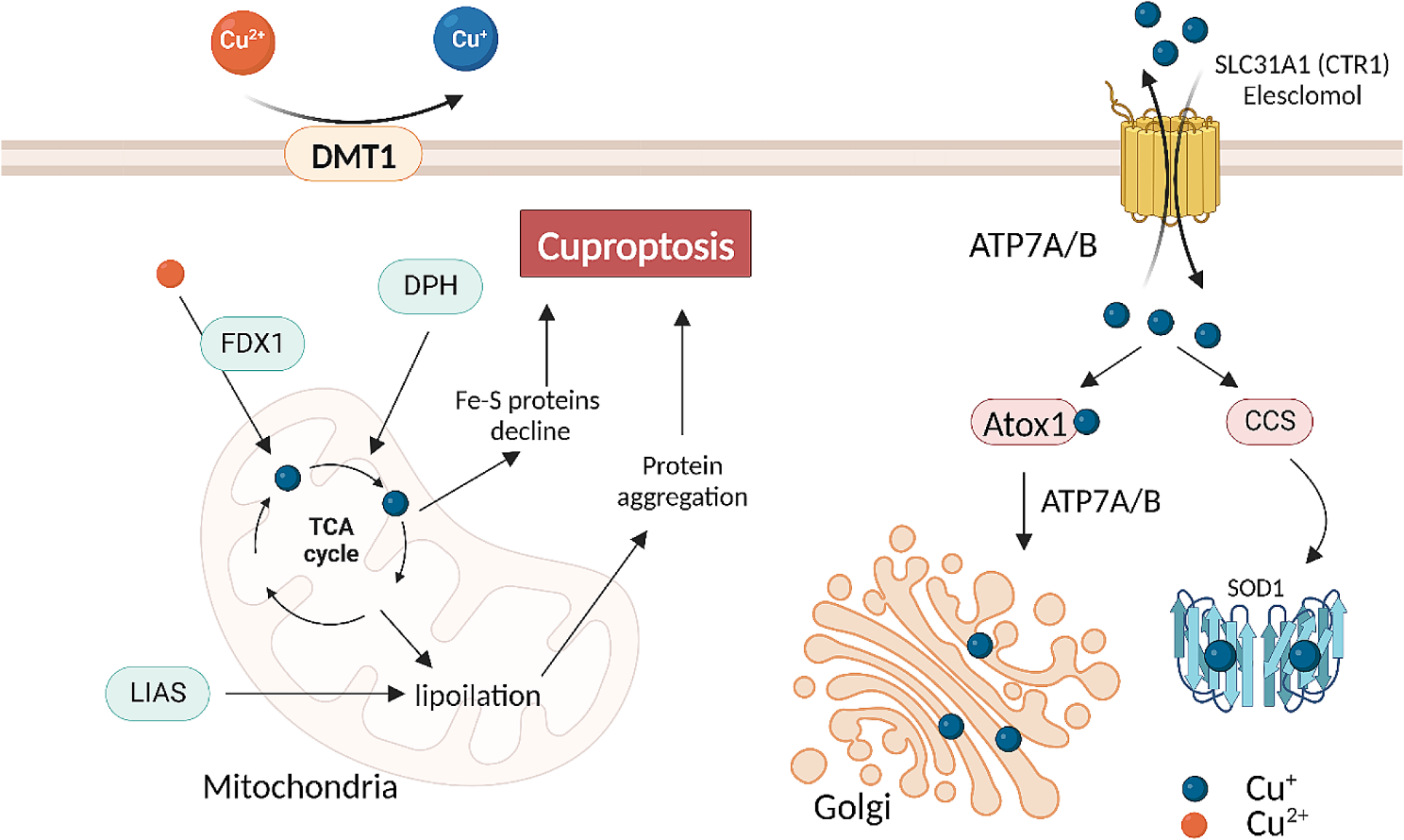
The regulation of intracellular Cu^+^. DMT1 reduces Cu^2+^ to Cu^+^. CTR1 (SLC31A1) deliver Cu^+^ to the cell cytoplasm and ATP7A/B export Cu+. CCS has got a copper binding motif and can deliver the metal element to SOD1. Intracellular copper binds the copper chaperone protein Atox1, which can form a complex with the copper transport ATP7A/B in the Golgi complex. Abbreviations: DMT1: Divalent metal transporter 1; CTR1: copper transporter 1; SLC31A1: solute carrier family 31 member 1; ATP7A/B: adenosine triphosphatase (ATPase) 7 A/B; CCS: Copper chaperone for superoxide dismutase; SOD: superoxide dismutases; Atox1: antioxidant 1

Knockout of FDX1 and/or the inhibition of lipoylation block cuproptosis [66]. An enzyme of the lipoic acid pathway, LIAS generates antioxidants in mitochondria [68]. LIPT1 and DLD also represent enzymes of the lipoic acid pathway and participate in protein lipoylation, required for activation of cuproptosis [69]. CTR1/SLC31A1 and ATP7A/B, the copper transporter, are required for intracellular transport of the element, the trigger of cytotoxicity [70]. ATP7A/B copper transporter is responsible for the regulation of normal physiological processes, such as the reabsorption of hepatic bile acids and the downregulation of intracellular concentration of copper and its toxicity (Fig. 1).

Copper ions can bind SOD1, a key defense enzyme against oxidative stress, ROS-related toxicity, membrane lipid peroxidation, and DNA damage [71]. Therefore, the excess of copper ions is associated with the increased oxidative damage. SOD enzymes transform the anion superoxide into hydrogen peroxide and are represented by three isoforms, including SOD1 (Cu/Zn dimeric form), SOD2 (mitochondrial tetrameric manganese (Mn) isoform), and SOD3 (extracellular tetrameric isoform) [72]. SOD1 expression and functioning deviate in different pathologies [73]. The activity of SOD1 is supported by a copper chaperone protein in the cell cytoplasm. Copper chaperone for superoxide dismutase (CCS) has got a copper-binding motif and delivers the metal element to SOD1. CCS also regulates the HIF-1 transcriptional complex and promotes the expression of vascular endothelial growth factor (VEGF), a tumor-stimulating effector [74]. Therefore, the role of copper in the SOD pathway is controversial and potentially may be associated with the development of cancer resistance [75].

In conclusion, the identified copper-induced genes play an important role during the activation of oxidative stress and the generation of ROS. Considering the sensitivity of various cancers to ROS-related toxicity, the identification of crosstalk targets between ROS- and copper-induced genes and their protein products in different cancers is warranted. The cuproptosis-related targets may be explored for the development of novel anti-cancer regimens. Recently, several new copper-triggered genetic targets were found activated in non-cancer pathologies. The activation of cuproptosis was reported in damaged brain cells (Alzheimer’s disease) and marked by the induction of various genes, including the gamma interferon-inducible lysosomal thiol reductase (GILT; encoded by tryptophane-tRNA ligase, cytoplasmic (IFI30) gene), phospholipase A1 member A (PLA1A), and arachidonate 5-lipoxygenase-activating protein (ALOX5AP) [14]. However, the role of copper in activation of these genes in brain cancer cells requires experimental confirmation.

## TME and cancer immunoediting are influenced by cuproptosis

TME consists of two major groups of cells, including heterogenous tumor cells and normal cells, such as various immune, endothelial (vasculature), and cells of local tissues (for instance, fibroblasts). All those cells may release a diverse range of signaling molecules, products of cell metabolism, and substances required for the maintenance of extracellular matrix (for solid tumors) and/or metastasis [76]. TME is transformed and activated during tumorigenesis, tumor spreading, and the development of drug resistance [77, 78]. Notably, TME cells can release anti-cancer effectors, although cancer cells manage to adapt and reverse the apoptosis-activating signaling. The process involves the dynamic interaction of cancer cells with surrounding tissue and the immune system. The complex crosstalk and resulting transformations were defined as cancer immunoediting which often leads to the cancer-promoting modifications of the immune system [79, 80]. During immunoediting, the immune cells (such as tumor-associated macrophages (TAM), NK cells, T and B lymphocytes), the key components of TME which are supposed to kill cancer cells and control tumor growth and spreading [81], are reprogrammed to promote or neglect carcinogenesis [82]. The molecular mechanisms of this transformation are complex and have been reviewed elsewhere [79, 80, 83]. In this review, we focus on the TME effectors which are targeted by cuproptosis, including TAM and NK cells.

During normal development of immune responses, macrophages differentiate into M1 and M2 types, where M1 is mainly involved in the activation of inflammatory response (pro-inflammatory type) [84]. M1 cells are programmed to promote inflammation and anti-tumor activities of other immune cells. Type M2 cells are involved in tissue repair and can suppress inflammation. M2 effectors help tumor cells escape the immune surveillance (pro-tumoral type of macrophages) [85]. The cells also secrete growth factors that stimulate tumor growth [86]. Both M1 and M2 cells were detected in TME [87, 88].

The sensitivity of M2 macrophages to cuproptosis was regulated by serine protease inhibitor clade E member 1 (SERPINE1) as demonstrated recently in gastric cancer [89]. In glioblastoma cells, retinoic acid receptor (RAR) responder (RARRES) was involved in regulation of the macrophage infiltration and is considered as a potential therapy response marker [90]. The expression level of cyclin-dependent kinase inhibitor 2 A (CDKN2A) was linked to cuproptosis in M2 cells in pulmonary fibrosis [91], although its role in lung cancer remains to be determined. The list of the genetic effectors associated with macrophage signaling is growing and was recently appended by a group of targets in alcohol-damaged liver cells [92]. The activation of these genes in liver cancer cells and TME was not tested.

NK cells (also known as tumor-infiltrating natural killer cells (TINKs)), powerful TME effectors, play an anti-tumor role in most solid tumor tissues [93, 94]. NK cell metabolic pathway can be changed during immunoediting which is marked by reduced cytotoxicity, inhibition of T cell growth and maturation, and ineffective cancer cell elimination [95]. CD4^+^T cells express programmed death 1 (PD-1) receptors, while tumor cells express the ligand to these receptors, PD-L1 [96]. Advanced cancer-infiltrating and killing abilities of NK, CD8+ T cells, and neutrophils were associated with FDX1 expression [59] . However, the expression of another cuproptosis-related gene SLC31A1 was negatively correlated with dendritic and NK cell infiltration in brain cancer [40,52, 59]. The binding of PD-1 and PD-L1 was reported to stimulate T-cell exhaustion and reduce the killing capacity of NK cells. This observation was used to develop anti-PD-L1 therapies [97]. A natural NK inhibitor, anti-cytotoxic T lymphocyte-associated antigen 4 (CTLA-4), is expressed on the surface of regulatory T cells and can suppress the immune response of NK cells [98]. Immune checkpoint inhibitors block these effectors, exerting an anti-tumor effect (described elsewhere) [98]. Combined application of cuproptosis-activating agents and anti-PD-L1 or immune checkpoint inhibitors represent a potential area for future investigations.

Other types of immune cells are also involved in the regulation of TME responses and have the potential to influence cuproptosis. B lymphocytes can act as an anti-tumor agent and secret tumor-specific antibodies [99]. Dendritic cells orchestrate and shape diverse TME responses during carcinogenesis [100]. A handful of recent research studies indicated the activation of B [101] and dendritic cells [102] during cuproptosis. However, findings are limited and require experimental confirmation. Regulatory roles and signaling effectors in B and dendritic cells during induction of cuproptosis remain unclear and warrant future investigations.

## Cuproptosis-related genes in hepatocellular carcinoma (HCC)

HCC ranks sixth in the incidence and second in the mortality of all cancers, representing a serious health burden worldwide [103]. Successful HCC treatment is complicated by limited surgical options, metastasis, and the development of chemotherapy resistance [104]. Therefore, the role of cuproptosis, as a potential new target for HCC treatment has been explored (Table 1). The analysis of cuproptosis-related gene expression indicated that FDX1, dihydrolipoamide dehydrogenase (DLD), and pyruvate dehydrogenase E1 subunit A1 (PDHA1) are positive regulators of this copper-associated PCD type in HCC [105]. Expression levels of lipoyltransferase 1 (LIPT1), dihydrolipoamide S-Acetyltransferase (DLAT), metal-regulatory transcription factor 1 (MTF1), glutaminase (GLS), and cyclin dependent kinase inhibitor 2 A (CDKN2A) genes were higher in HCC patients [105, 106]. The survival time of HCC patients with high expression of FDX1 was prolonged, while the poorer prognosis of HCC patients with low expression of PDX1 was indicated [105]. The overall survival (OS) of HCC patients with higher expression levels of GLS, DLAT, and CDKN2A was lower, compared to the patients with decreased expression levels of these genes [107]. To confirm these observations, all findings related to gene expression level should be verified at the level of protein expression.

**Table 1:** Differential expression levels of cuproptosis-related genes in malignant tissues

Gene/ protein	HCC	LC	GC	BCa	KIRC	Major functions	Ref.
Ferredoxin 1 (**FDX1**)	$\downarrow$	$\downarrow$	$\uparrow$	$\downarrow$	$\downarrow$	• Transfer of electrons from NADPH to mitochondrial cytochrome P450; • Regulation of glucose metabolism shift from glycolysis to mitochondrial respiration; • Regulation of the biosynthesis of heme A, Fe/S clusters and steroidogenesis.	[66, 107, 120]
Lipoic acid synthetase (**LIAS**)	$\uparrow$	$\uparrow$	$\uparrow$	$\downarrow$	$\downarrow$	• Synthesis of lipoic acid by introducing two sulfhydryl groups at the C6 and C8 sites of the octanoic acid moiety.	[144–146]
Lipoyltrans-ferase 1 (**LIPT1**)	$\uparrow$	$\uparrow$	$\uparrow$	$\downarrow$	$\downarrow$	• Activation of TCA cycle-associated 2-ketoacid dehydrogenases; • Fatty acyltransferase 1/ regulation of lipoic acid (LA) transport; • Catalysis of the transfer of a lipoyl group to the lysine residue of the target proteins.	[69, 105, 144]
Dihydrolipoamide dehydrogena-se (**DLD**)	$\uparrow$	$\uparrow$	$\uparrow$	$\downarrow$	$\downarrow$	• Third catalytic enzyme of the pyruvate dehydrogenase complex (PDHC), involved in TCA cycle by converting pyruvate to acetyl coenzyme A (acetyl-CoA); • E3 component of PDC which acts as dihydrolipoamide dehydrogenase and catalyzes the formation of NADH.	[115, 124, 144, 146]
Drolipo-amide S-acetyltransferase (**DLAT**)	$\uparrow$	$\uparrow$	$\uparrow$	$\uparrow$	$\downarrow$	• E2 essential subunit of PDHC; • E2 component of PDC that acts as a dihydrolipoamide acetyltransferase during the biosynthesis of acetyl-CoA.	[69, 144]
Pyruvate dehydrogena-se E1 subunit alpha 1 (**PDHA1**)	$\uparrow$	$\uparrow$	$\uparrow$	$\downarrow$	$\downarrow$	• α1 subunit of the PDC E1 component, • Regulation of pyruvate dehydrogenase mediating pyruvate decarboxylation.	[144]
Pyruvate dehydrogena-se E1 subunit beta (**PDHB**)	$\uparrow$	$\uparrow$	$\uparrow$	$\uparrow$	$\downarrow$	• β subunit of the PDC E1 component, • Regulation of pyruvate dehydrogenase mediating pyruvate decarboxylation.	[124, 144]
Metal-regulatory transcription factor-1 (**MTF1**)	$\uparrow$	$\downarrow$	$\uparrow$	$\downarrow$	$\downarrow$	• Activation of the transcription of copper binding protein MT, by binding to the metal response element in MT promoter.	[124, 144]
Glutaminase (**GLS**)	$\uparrow$	$\downarrow$	$\uparrow$	$\downarrow$	$\downarrow$	• Regulation of the catabolism of glutamine, conversion of glutamine into glutamate, maintenance of the glutamate homeostasis.	[144]
Cyclin-dependent kinase inhibitor 2 A (**CDKN2A**)	$\uparrow$	$\uparrow$	$\uparrow$	$\uparrow$	$\uparrow$	• Cell cycle arrest at G1 and G2 phases; • Inhibition of the oncogenic effects of CDK4/6 and MDM2.	[69, 144]
Adenosine triphosphate-se (ATPase) 7 A/B (**ATP7A/B**)	$\uparrow$	$\downarrow$	$\uparrow$	$\uparrow$	$\uparrow$	• Transfer of Cu^+^ across the membrane from delivery to acceptor proteins without establishing a free copper gradient; • Regulation of Cu^+^ exit from cells.	[2, 65, 144, 145]
Solute carrier family 31 member 1 (**SLC31A1**)	**-**	$\uparrow$	$\uparrow$	$\uparrow$	$\downarrow$	• Regulation of copper entry into cells.	[19, 144, 145]

Abbreviations: Upregulation of a gene expression is indicated by ↑; while downregulation is indicated by ↓. Hepatocellular carcinoma, HCC; Lung cancer, LC; Breast cancer, BCa; Kidney renal clear cell carcinoma, KIRC. The unknown (not tested) level is indicated by “-“

Patients with high expression of CDKN2A demonstrated higher levels of B cells, CD4^+^T cells, and macrophages in the TME [108]. Cuproptosis-related risk score (CRRS) indicated that patients with a high CRRS have a poorer prognosis, lower OS, increased matrix activity and immune infiltration, and are characterized by activation of abundant cancer pathways [108]. The immune escape of the high-risk group was prevented by immunotherapy, showing the important role of immunosuppression in TME [108]. At the same time, levels of immune checkpoint effectors in the high-risk score group were high and included high expression of PDCD1 and CTLA4. Therefore, immune checkpoint inhibitor (ICI) immunotherapy was effective [107]. However, the role of these genes and their protein targets in HCC-related cuproptosis warrants further investigation.

The human liver accumulates copper deposits in the protein-bound form as part of hepatic metallothionein (MT), a cysteine-rich molecule. A low-molecular-weight protein MT has a high affinity for metals [109]. MT protects against copper toxicity via the simple retaining of copper ions [110]. The role of MT in the activation of cuproptosis in HCC remains to be tested. Controversial findings were indicated for MT in HCC. Downregulated levels of MT1 were reported in HCC [111]. Expression levels of MT may indicate cancer cell responsiveness to cuproptosis, although it was not assessed in HCC patients. Interestingly, a recent study indicated that MT expression is increased after Lenvatinib therapy and associated with lowered survival of HCC patients [112].

## Cuproptosis-targeted genes in lung cancer

Lung cancer is a malignant tumor with very high incidence and mortality worldwide [113]. Early lung cancer diagnostics is poorly developed, reflecting the high heterogeneity and complexity of this type of cancer [114]. A recent study demonstrated the upregulation of seven genes (DLAT, DLD, glycine cleavage system protein H (a protein-coding gene, GCSH), LIAS, LIPT1, PDHA1, and PDHB) in lung adenocarcinoma patients (Table 1) [115]. During this study testing, three genes (ATP7B, FDX1, and SLC31A1) were found downregulated [115]. In lung cancer patients, high expression of DLD, DLAT, PHDA1, PHDB, and CDKN2A were associated with poor OS, while high expression of MTF1 was associated with longer OS [116]. In the high-risk group, immune cell infiltration was reduced [117]. Another recent study assessed the expression of cuproptosis-related genes during T-cell exhaustion [118].

In lung cancer stem cells, eight differently expressed genes (Krueppel-like transcription factor 4 (KLF4), secretoglobin family 3 A member1 (SCGB3A1), collagen type I alpha 1 chain (COL1A1), secreted phosphoprotein 1 (SPP1), Complement Component 4 Binding Protein Alpha (C4BPA), Tetraspanin 7 (TSPAN7), caveolin 2 (CAV2), and Collagen Triple Helix Repeat Containing 1 (CTHRC1); stemness gene signature) were tested and validated *in vitro* as markers to predict the lung cancer progression [118]. The study demonstrated that the expression of KLF4, COL1A1, SPP1, CAV2, and CTHRC1 positively correlated with the expression of immune checkpoint proteins, while TSPAN7, C4BPA, and PSMB9 showed a negative association [119]. Further validation of findings is warranted and should include the testing of relevant enzymatic activities.

## Cuproptosis-related genes in gastric cancer

FDX1 gene expression was found to be upregulated in gastric cancer patients, suggesting a pro-carcinogenic role of these proteins [120]. However, in another study, gastric cancer patients with high expression of the FDX1, LIAS, SLC31A1, DLAT, and ATP7A/B genes demonstrated a better prognosis (Table 1) [121]. The study also found that gastric cancer patients with high expression of DLST had a poor prognosis and reduced survival time [121]. To optimize analysis cuproptosis-related gene (CRG) expression signatures were generated and used as CRG scores [122–124]. Compared with the low CRG score group, the high CRG score group was marked by a worse prognosis, shorter survival, fewer immune checkpoint targets, and higher tumor-linked immune dysfunctions [124]. In patients with gastric cancer, a high CRG score was also associated with advanced M2 (macrophage) infiltration and NK cell activity [119]. In contrast to NK cells, the lower level of mast cells, an important innate immune cell component, was linked to the poorer survival of patients from the high CRG score group [125]. Interestingly, another study confirmed that the infiltration level of various immune cells (including NK cells, neutrophils, and macrophages M2) positively correlated with the expression of SERPINE1 (also known as plasminogen activator inhibitor-1, PAI-1) during activation of cuproptosis in gastric cancer cells [89]. Higher expression of SERPINE1/PAI-1 leads to poor cancer prognosis and the development of resistance to bevacizumab (anti-VEGF-based immunotherapy) [126]. The activation of cuproptosis in mast cells remains unexplored [127], although mast cells are a very promising target in TME. The role of mast cells in the regulation of TME was recently discussed elsewhere [128].

One of the most important components of anti-cancer immune responses, CD4^+^ memory T cells are required for successful cancer elimination. It has been shown that CD4^+^T cell infiltration correlated with better survival time and prognosis in gastric cancer patients [125]. The higher activation of CD4^+^T cells and plasma cells in TME was reported in patients with low CRG scores. The group was also marked by a better prognosis [121]. Recent analysis indicated that expression of cuproptosis-induced FDX1 negatively correlates with numbers of CD4 + T cells and cancer-associated fibroblasts (CAFs) infiltration [129, 130]. Anti-cancer benefits of CD4 + T-cell activation during immune surveillance have been discussed elsewhere [131, 132].

## Cuproptosis-related targets in breast cancer (BCa)

Expression of cuproptosis-related genes was assessed in BCa patients [133]. The analysis indicated a diversity of responses. For instance, up-regulated SLC31A1 levels correlated with poor OS, whereas high expression levels of LIPT1 and PDHA1 correlated with a better prognosis and OS [134]. In high-risk BCa patients, TME is marked by the expression of fewer immune checkpoint effectors, higher tumor stemness, and the presence of mainly resting macrophages (M2, M0) and NK cells. The profile of BCa patients with low CRG scores is characterized by abundant infiltration of immune cells, including anti-tumor lymphocytes, macrophages M1, CD8^+^T cells, and activated NK cells. Most of these cells expressed increased levels of PD-1, PD-L1, and CTLA4. Accordingly, the therapeutic effect of immune checkpoint inhibitors, as well as the sensitivity to immune therapy were higher in the BCa patients with increased infiltration of immune cells [133, 135, 136]. Table 1 summarizes the expression level of cuproptosis-related genes in BCa patients. However, the clinical application of cuproptosis as anti-BCa therapy tool remains to be tested.

## Cuproptosis targets in kidney renal clear cell carcinoma (KIRC)

KIRC is the most common type of renal cell carcinoma (RCC) [137]. KIRC is characterized by a poor prognosis as it is often diagnosed at an advanced stage and patients develop resistance to radio- and chemotherapy [138]. Cuproptosis-related genes in patients with KIRC were analyzed and correlated with clinical parameters, diagnosis, and prognosis. KIRC patients with high expression of FDX1 demonstrated a good prognosis and high OS rate (Table 1) [139]. The low-risk CRG score positively correlated with the infiltration of macrophages, monocytes, CD8^+^T cells, and Tregs, whereas the high-risk CRG score negatively correlated with the infiltration of neutrophil, NK cells, and non-regulatory CD4^+^T cells. The immunotherapy responses and prognosis in the high-risk group were poorer than that of the low-risk group [140]. However, the expression of various cuproptosis-related genes and their targets in RCC remains to be confirmed. For instance, a recent study, which assessed levels of FDX1, found this gene highly expressed in 15 different tumors, while the gene was downregulated in 11 other tumors [141], suggesting highly differential expression of this cuproptosis marker. The same group identified FDX1-linked enrichment of genes in KIRC, including the tricarboxylic acid (TCA) cycle, NOTCH pathway, and others [141]. Many of these pathways are being explored as promising anti-RCC targets [142, 143].

We constructed Table 2 to summarize the information about current and completed cancer-targeting clinical trials which tested the role of cuproptosis.

**Table 2:** Clinical trials for cuproptosis-regulating compounds

NCT Number	Compound/ Drug	Phase	Patient #	Status	Cancer type	Title	Conclusion	Ref.
NCT00522834	Elesclomol (STA-4783)/ Paclitaxel	Phase 3	651	terminated	Melanoma	Elesclomol (STA-4783) with Paclitaxel Versus Paclitaxel Alone in Melanoma	The addition of elesclomol to paclitaxel did not significantly improve PFS, combination therapy improved PFS in patients with normal serum LDH levels.	[147]
NCT01280786	Elesclomol (STA-4783)	Phase 1	36	unknown status	Relapsed or Refractory Acute Myeloid Leukemia	Study Elesclomol Sodium in Patients with Relapsed or Refractory Acute Myeloid Leukemia	N/A	*
NCT00827203	Elesclomol STA-4783	Phase 1	30	suspended	Solid Tumors	A Safety Study to Determine the Maximum Tolerated Dose of Elesclomol Sodium in Patients with Solid Tumors	The STA-4783/paclitaxel combination was well tolerated with a toxicity profile similar to single-agent paclitaxel.	[148]
NCT00888615	Elesclomol (STA-4783)/ Paclitaxel	Phase 2	58	completed	Ovarian Epithelial Cancer, Fallopian Tube Cancer, or Primary Peritoneal Cancer	Elesclomol Sodium and Paclitaxel in Treating Patients with Recurrent or Persistent Ovarian Epithelial Cancer, Fallopian Tube Cancer, or Primary Peritoneal Cancer	This combination was well tolerated but is unworthy of further investigation.	[149]
NCT00808418	Elesclomol (STA-4783) Docetaxel and Prednisone	Phase 1	34	completed	Metastatic Prostate Cancer	A Study to Determine the Maximum Tolerated Dose of Elesclomol Sodium Given with a Fixed Dose of Docetaxel and Prednisone in Patients with Metastatic Prostate Cancer	N/A	*
NCT00087997	Elesclomol (STA-4783) Paclitaxel	Phase 2	80	completed	Soft Tissue Sarcomas	A Study of STA-4783 in Combination with Weekly Paclitaxel for Treatment of Patients with Soft Tissue Sarcomas	N/A	*
NCT00088114	Elesclomol (STA-4783) Paclitaxel	Phase 1	50	completed	Solid Tumors	STA-4783 and Paclitaxel for Treatment of Solid Tumors	N/A	*
NCT00084214	Elesclomol (STA-4783) Paclitaxel	Phase 1 Phase 2	103	completed	Melanoma	STA-4783/​Paclitaxel or Paclitaxel Alone in Melanoma	E + P resulted in a statistically significant doubling of median PFS, with an acceptable toxicity profile and encouraging OS	[150]
NCT00088088	Elesclomol (STA-4783) Paclitaxel and Carboplatin	Phase 1 Phase 2	86	completed	Stage IIIB or Stage IV Non-Small Cell Lung Cancer (NSCLC)	STA-4783 in Combination with Paclitaxel and Carboplatin for the Treatment of Chemotherapy Naive Patients with Stage IIIB/​IV Non-Small Cell Lung Cancer (NSCLC)	N/A	*
NCT02715609	DSF/Copper Radiation Therapy and Temozolomide	Phase 1 Phase 2	35	active, not recruiting	Newly Diagnosed Glioblastoma	Disulfiram/​Copper with Concurrent Radiation Therapy and Temozolomide in Patients with Newly Diagnosed Glioblastoma	N/A	*
NCT00256230	DSF	Phase 1 Phase 2	7	completed	Metastatic Melanoma	Disulfiram in Patients with Metastatic Melanoma	N/A	*
NCT03363659	DSF Temozolomide	Phase 2	15	terminated	Unmethylated Glioblastoma Multiforme	Disulfiram and Copper Gluconate with Temozolomide in Unmethylated Glioblastoma Multiforme	N/A	*
NCT05210374	DSF Copper Gluconate and Liposomal Doxorubicin	Phase 1	24	recruiting	Treatment-Refractory Sarcomas	Disulfiram with Copper Gluconate and Liposomal Doxorubicin in Treatment-Refractory Sarcomas	N/A	*
NCT03714555	DSF-Copper Gluconate Abraxane-Gemzar, FOLFIRINOX or Gemcitabine	Phase 2	1	terminated	Met Pancreas Cancer	Disulfiram-Copper Gluconate in Met Pancreas Cancer with Rising CA19-9 on Abraxane-Gemzar, FOLFIRINOX or Gemcitabine	N/A	*
NCT03034135	DSF and Copper Gluconate Temozolomide	Phase 2	23	completed	Recurrent Glioblastoma	Safety, Tolerability and Efficacy of Disulfiram and Copper Gluconate in Recurrent Glioblastoma	Addition of DSF/Cu to TMZ for TMZ-resistant IDH-wild type GBM appears well tolerated but has limited activity for unselected population.	[151]
NCT03323346	DSF	Phase 2	##	recruiting	Metastatic Breast Cancer	Phase II Trial of Disulfiram with Copper in Metastatic Breast Cancer (DISC)	N/A	*
NCT03950830	DSF Cisplatin	Phase 2	12	completed	multiple relapsed/refractory germ cell tumors (GCTs)	Disulfiram and Cisplatin in Refractory TGCTs. (DISGCT)	N/A	*
NCT01118741	DSF	N/A	19	completed	Recurrent Prostate Cancer with Rising Prostate Specific Antigen (PSA)	Study of Recurrent Prostate Cancer with Rising Prostate Specific Antigen (PSA)	DSF treatment is tolerated but has no clinical benefit	[152]
NCT00312819	DSF Standard Chemotherapy	Phase 2 Phase 3	60	completed	Lung Cancer	Initial Assessment of the Effect of the Addition of Disulfiram (Antabuse) to Standard Chemotherapy in Lung Cancer	N/A	[153]
NCT00571116	DSF Arsenic Trioxide	Phase 1	9	terminated	Metastatic Melanoma	Disulfiram Plus Arsenic Trioxide in Patients with Metastatic Melanoma and at Least One Prior Systemic Therapy	N/A	*
NCT01777919	DSF/​Copper Temozolomide	Phase 2	32	unknown status	Newly Diagnosed Glioblastoma Multiform (GLIODIS)	Disulfiram/​Copper Combination in The Treatment of Newly Diagnosed Glioblastoma Multiform (GLIODIS)	N/A	[154]
NCT04521335	DSF Copper Gluconate	Phase 1	2	terminated	Treatment-Refractory Multiple Myeloma (Repurpose-1)	Study of Disulfiram and Copper Gluconate in Patients with Treatment-Refractory Multiple Myeloma (Repurpose-1)	N/A	[155]
NCT00742911	DSF Copper Gluconate	Phase 1	21	completed	Refractory Solid Tumors Involving the Liver	Phase I Study of Disulfiram and Copper Gluconate for the Treatment of Refractory Solid Tumors Involving the Liver	Disulfiram 250 mg daily with copper gluconate (8 mg of elemental copper) was well-tolerated in patients with solid tumors involving the liver and was not associated with dose limiting toxicities.	[156]
NCT04265274	DSF Vinorelbin, Cisplatin, Copper	Phase 2	0	withdrawn	CTC_EMT Positive Refractory Metastatic Breast Cancer	Vinorelbine, Cisplatin, Disulfiram and Copper in CTC_EMT Positive Refractory Metastatic Breast Cancer.	N/A	*
NCT05667415	DSF cisplatin	N/A	40	not yet recruiting	Gastric Cancer	Treatment of Advance Gastric Cancer with Disulfiram	N/A	*
NCT03363659	DSF Copper gluconate, Temozolomide	Phase 2	15	terminated	Unmethylated Glioblastoma Multiforme	Disulfiram and Copper Gluconate with Temozolomide in Unmethylated Glioblastoma Multiforme	N/A	*
NCT02678975	DSF Copper, Alkylating Agents	Phase 2 Phase 3	88	completed	Recurrent Glioblastoma	Disulfiram in Recurrent Glioblastoma	DSF combined with Alkylating treatment has limited risk profile	[157]
NCT02963051	DSF Copper, Copper gluconate	Phase 1	9	terminated	Metastatic, Castration Resistant Prostate Cancer	A Phase Ib Study of Intravenous Copper Loading with Oral Disulfiram in Metastatic, Castration Resistant Prostate Cancer	N/A	*
NCT01907165	DSF Copper gluconate, Temozolomide	Early Phase 1	21	completed	Glioblastoma Multiforme	Disulfiram in Treating Patients with Glioblastoma Multiforme After Radiation Therapy with Temozolomide	Disulfiram can be safely combined with temozolomide but can cause reversible neurological toxicities.	[158]
NCT05210374	DSF Copper gluconate, Liposomal Doxorubicin (Doxil)	Phase 1	24	recruiting	Treatment-Refractory Sarcomas	Disulfiram with Copper Gluconate and Liposomal Doxorubicin in Treatment-Refractory Sarcomas	N/A	*
NCT02671890	DSF Chemotherapy, Gemcitabine Hydrochloride	Phase 1	16	active, not recruiting	Refractory Solid Tumors or Metastatic Pancreatic Cancer	Disulfiram and Chemotherapy in Treating Patients with Refractory Solid Tumors or Metastatic Pancreatic Cancer	N/A	*
NCT02715609	DSF Copper Gluconate, Concurrent Radiation Therapy and Temozolomide	Phase 1 Phase 2	35	active, not recruiting	Newly Diagnosed Glioblastoma	Disulfiram/​Copper with Concurrent Radiation Therapy and Temozolomide in Patients with Newly Diagnosed Glioblastoma	N/A	*
NCT02770378	DSF Temozolomide, Aprepitant, Minocycline, Celecoxib	Phase 1 Phase 2	10	completed	Recurrent Glioblastoma	A Proof-of-concept Clinical Trial Assessing the Safety of the Coordinated Undermining of Survival Paths by 9 Repurposed Drugs Combined with Metronomic Temozolomide (CUSP9v3 Treatment Protocol) for Recurrent Glioblastoma	Nine drug combinations, including DSF, can be applied safely with careful monitoring	[159]

* The information and clinical trial number were found using the publicly available search tool at https://www.clinicaltrials.gov/

Unknown (not published) information is indicated by “N/A” (not applicable)

## Cuproptosis as a novel immunotherapy target

The success of anti-cancer therapy is obstructed by delayed diagnosis, recurrence, poor prognosis, and limited treatment methods. However, the development of targeted (personalized) therapy and combined immunotherapy methods delivers promising results. Malignant cells can evade the attack of immune cells and develop cancer tolerance through the transformation of immune checkpoint signaling. The most widely studied immune checkpoint effectors are cytotoxic T lymphocyte-associated antigen 4 (CTLA-4) and PD-1. Application of ICI can reverse immune tolerance and reactivate T cell-mediated cytotoxicity (anti-tumor effects) [160]. The combined regimen with ICI and bevacizumab demonstrated effectiveness in the treatment of HCC [161]. Anti-cancer effects of cuproptosis and the discovery of cuproptosis-related genes and proteins open new horizons in cancer therapy. The tumor-associated antigens, released during activation of cuproptosis, are recognized by immune system, can activate immune responses, and enhance the efficacy of current immunotherapies. Accordingly, cuproptosis-activating agents can complement the existing immunotherapy drugs and potentially provide a stronger anti-cancer response. Targeted activation of cuproptosis pathways may favor the re-activation of TME towards the eradication of cancer cells [89, 162–164]. However, the mechanisms of the anti-cancer effects of agents which can modulate copper metabolism and signalling in malignant tissues remain to be investigated

**Fig. 2:**
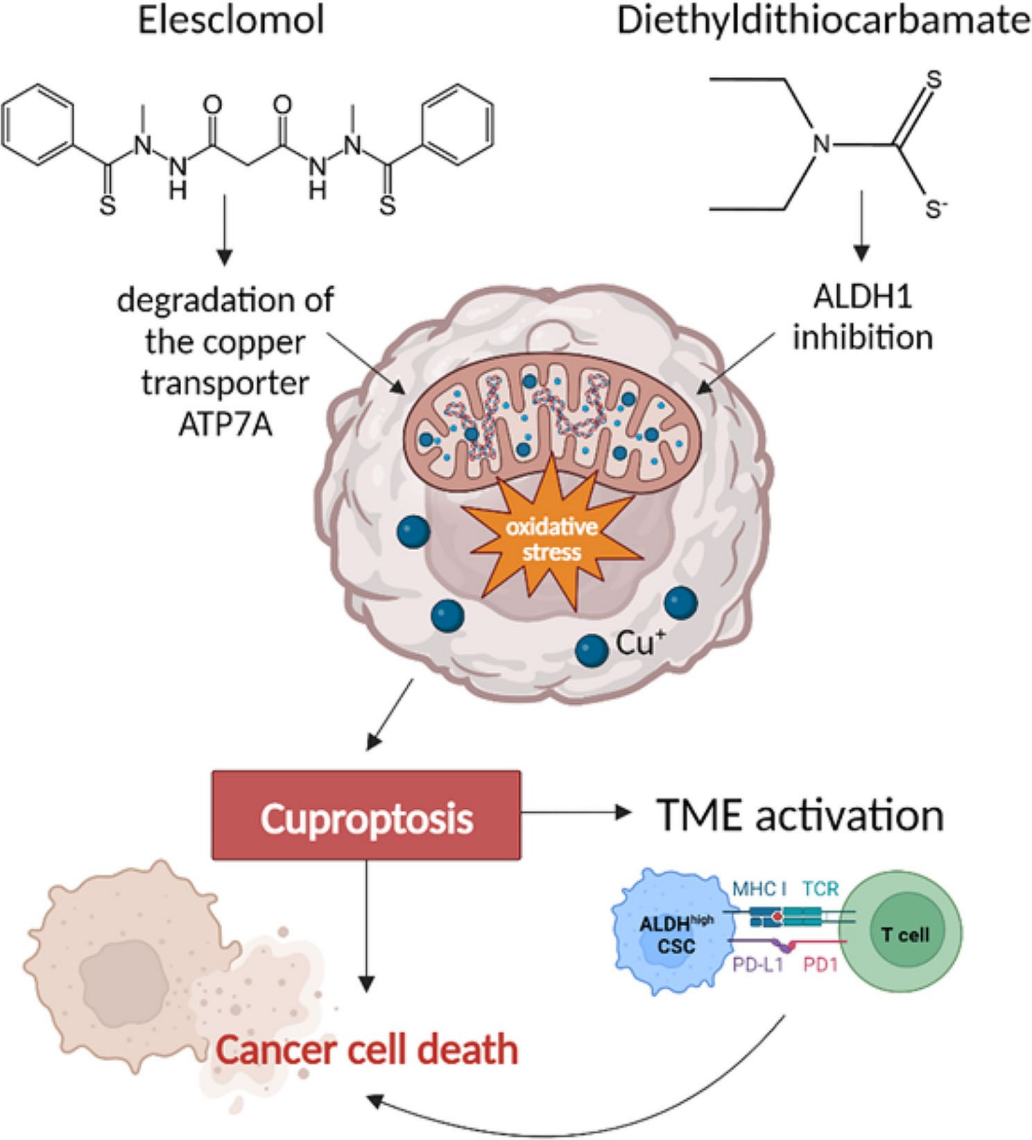
A promising therapeutic strategy for triggering the immune response in tumor cells based on the induction of cuproptosis by Elescomol or Diethyldithiocarbamate. Abbreviations: ATP7A: Menkes ATPase; a proliferation-regulating effector; ALDH1: aldehyde dehydrogenase 1; TME: tumor microenvironment

There are several methods to control copper signaling. To regulate the concentration of copper in cells, copper chelators were generated and designed to reduce the bioavailability of copper [165]. To regulate intracellular copper transport, there are copper ionophores that can be used to increase the intracellular concentration of copper ions and trigger cytotoxic stress [166]. However, these drugs lack specificity and selectivity. For instance, elesclomol (a copper ionophore) can deliver copper ions to the cytosol, increase the intracellular concentration of copper ions, promote oxidative stress, and induce cuproptosis [4, 167]. Elesclomol demonstrated promising therapeutic effects in the treatment of several diseases and cancer [167] (Fig. 2)

Interestingly, elesclomol stimulated the degradation of the copper transporter ATP7A in intestinal cancer cells and increased the concentration of copper in cancer mitochondria [168]. Suggestively, the combination of elesclomol and other chemotherapeutic drugs can improve anti-cancer efficacy. This hypothesis remains to be tested. Another copper ion carrier diethyldithiocarbamate (the active metabolite of disulfiram, an inhibitor of aldehyde dehydrogenase 1 (ALDH1) [169]) was also shown to increase intracellular copper concentrations [170]. It has been suggested that disulfiram may be used for the treatment of a variety of cancers, including colorectal and breast cancers [171]. We summarize the information about clinical trials of copper transporters in Table 2.

Copper transporter ATP7A/B has been found to mediate chemotherapeutic cancer resistance. High expression of ATP7A/B was observed in cells resistant to platinum-based chemotherapeutic drugs. Accordingly, silencing of ATP7A/B increased the sensitivity to chemotherapy [172]. As a copper transporter, ATP7A/B is an important effector of cuproptosis in cancer cells [173]. Although the mechanism of ATP7A/B signaling in tumors remains unclear, the transporter was indicated as a potential anti-cancer target and/or therapy response marker [145]. The hypothesis warrants future clinical testing.

To enhance the efficacy of cancer treatment, chemotherapy/immunotherapy regimens may be tested in combination with several copper-carriers which are in clinical trials (Table 2). Aside from immunotherapy, ferroptosis-inducing substances were found to enhance cuproptosis in liver cancer cells [174], suggesting a potential additive or synergistic effect. Whether the combination of ferroptosis and cuproptosis-targeting agents can improve the effect of cancer treatment remains to be confirmed. Notably, the application of combined multi-component therapies may be associated with higher risks. There is no reliable cancer-targeting delivery system of copper ions. It is also unclear how to estimate and maintain a less harmful level of copper in normal cells and in vital organs. Effects of copper-transporting systems in many normal cells remains under-addressed, suggesting a potential risk of copper-toxicity during combined therapies. Interdisciplinary approach should be employed to clarify the systemic toxicity of copper-transporting agents. Selective induction of cuproptosis alone and combined with other anti-cancer treatment regimens in cancer cells warrants future investigations.

## Conclusions and future perspectives

Copper signaling represents an attractive therapeutic target, although both beneficial and toxic copper-induced effects were reported. Copper is an essential microelement that is required for normal physiological functions [2, 4]. However, dysbalanced copper metabolism was linked to the progression of various diseases, including cancer [175]. The discovery of cuproptosis, a new form of cell death [21, 146], uncovered a relatively new copper-associated mechanism of signaling and a new anti-cancer target. 

Several recent studies reported discovery of copper-targeted genes associated with cuproptosis, including FDX1, LIAS, LIPT1, DLD, DLAT, PDHA1/B, MTF1, GLS, CDKN2A, ATP7A/B, SLC31A1 [29, 65, 66, 69, 70, 105–108]. Differential expression of these genes in malignant and normal tissues has been shown, suggesting their involvement in the regulation of carcinogenesis. Furthermore, the expression of cuproptosis-related genes correlated with TME characteristics and disease prognosis. For instance, levels of FDX1 were decreased in many cancers and reflected the level of immune cell infiltration [59,176]. However, functional implications of the differential expression of cuproptosis-related genes in normal vs. cancer cells remains to be clarified.

Successful cancer immunotherapy is obstructed by the development of immune tolerance and the escape of tumor cells from immune surveillance, defined as cancer immunoediting [177]. Cuproptosis and cuproptosis-related genes represent a novel anti-cancer target that can be also employed for the anti-cancer priming of TME [144] and re-activating of natural anti-cancer surveillance. Targeted activation of cuproptosis-related genes may be tested as a priming or contributing factors for improving current ICI immunotherapy. However, the molecular mechanism of cuproptosis and its clinical safety warrant future investigations and clinical validations.

## Data Availability

No datasets were generated or analysed during the current study.

## Abbreviations

ALOX5AP: Arachidonate 5-lipoxygenase-activating protein
Atox1: Antioxidant 1
ATP7A: Adenosine triphosphatase (ATPase) 7A /Menkes ATPase
ATP7B: Adenosine triphosphatase (ATPase) 7B/ATPase7B
BCa: Breast cancer
C4BPA: Complement Component 4 Binding Protein Alpha
CAFs: Cancer-associated fibroblasts
CAV2: Caveolin 2
CCS: Copper chaperone for superoxide dismutase
CDK4/6: Cyclin dependent kinases 4 and 6
CDKN2A: Cyclin-dependent kinase inhibitor 2A
COL1A1: Collagen type I alpha 1 chain
COX: Cytochrome c oxidase
CRG: Cuproptosis-related gene
CRRS: Cuproptosis-related risk score
CTHRC1: Collagen triple helix repeat containing 1
CTLA-4: Anti-cytotoxic T lymphocyte associated antigen 4
CTR1: Copper transporter 1
DLAT: Drolipoamide S-acetyltransferase
DLD: Dihydrolipoamide dehydrogenase
DMT1: Divalent metal transporter 1
Fe-S cluster protein: Iron-sulfur cluster protein
FDX1: Ferredoxin 1
GC: Gastric cancer
GCSH: Glycine cleavage system protein H
GILT: Gamma interferon-inducible lysosomal thiol reductase
GLS: Glutaminase
HCC: Hepatocellular carcinoma
ICI: Immune checkpoint inhibitor
KIRC: Kidney renal clear cell carcinoma
KLF4: Krueppel-like transcription factor 4
LA: Lipoic acid
LC: Lung cancer
LIAS: Lipoic acid synthetase
LIPT1: Lipoyltransferase 1
MDM2: Murine double minute
MT: Metallothionein
MTF1: Metal-regulatory transcription factor-1
NADPH: Nicotinamide adenine dinucleotide phosphate
ND2: NADH deoxygenase-2
OS: Overall survival
PD-1: Programmed death 1
PDC: Pyruvate dehydrogenase complex
PDHA1: Pyruvate dehydrogenase E1 subunit alpha 1
PDHB: Pyruvate dehydrogenase E1 subunit beta
PDHC: Pyruvate dehydrogenase complex
PD-L1: Programmed death 1 receptors
PLA1A: Phospholipase A1 member A
RAR: Retinoic acid receptor
RARRES: Retinoic acid receptor responder
ROS: Reactive oxygen species
SCGB3A1: Secretoglobin family 3A member1
SERPINE1: Serine protease inhibitor clade E member 1
SLC31A1: Solute carrier family 31 member 1
SODs: Superoxide dismutases
SPP1: Secreted phosphoprotein 1
TAM: Tumor associated macrophages
TCA: Tricarboxylic acid
TINKs: Tumor infiltrating natural killer cells
TME: Tumor microenvironment
TSPAN7: Tetraspanin 7
VEGF: Vascular endothelial growth factor
